# Signal Amplification by Enzymatic Reaction in an Immunosensor Based on Localized Surface Plasmon Resonance (LSPR)

**DOI:** 10.3390/s100302045

**Published:** 2010-03-12

**Authors:** Tae-Han Lee, Seung-Woo Lee, Ji-Ae Jung, Junhyoung Ahn, Min-Gon Kim, Yong-Beom Shin

**Affiliations:** 1 Korea Research Institute of Bioscience and Biotechnology (KRIBB), Daejeon 305-806, Korea; E-Mail: swlee71@kribb.re.kr (S.-W.L); 2 University of Science and Technology (UST), 305-333 Daejeon, Korea; E-Mails: t2taehan@gmail.com (T.-H.L.); jiae86@kribb.re.kr (J.-A.J.); ajh@kribb.re.kr (J.A); mgkim@kribb.re.kr (M.-G.K.)

**Keywords:** immunosensor, localized surface plasmon resonance (LSPR), gold nano-island, enzyme-catalyzed precipitation

## Abstract

An enzymatic reaction was employed as a means to enhance the sensitivity of an immunosensor based on localized surface plasmon resonance (LSPR). The reaction occurs after intermolecular binding between an antigen and an antibody on gold nano-island (NI) surfaces. For LSPR sensing, the gold NI surface was fabricated on glass substrates using vacuum evaporation and heat treatment. The interferon-γ (IFN-γ) capture antibody was immobilized on the gold NIs, followed by binding of IFN-γ to the antibody. Subsequently, a biotinylated antibody and a horseradish peroxidase (HRP) conjugated with avidin were simultaneously introduced. A solution of 4-chloro-1-naphthol (4-CN) was then used for precipitation; precipitation was the result of the enzymatic reaction catalyzed the HRP on gold NIs. The LSPR spectra were obtained after each binding process. Using this method, the enzyme-catalyzed precipitation reaction on the gold NI surface was found to effectively amplify the change in the signal of the LSPR immunosensor after intermolecular binding.

## 1. Introduction

Studies on the detection of biomolecules using localized surface plasmon resonance (LSPR) have been recently accelerated by the development of various nanostructure fabrication techniques. In particular, label-free detection technologies based on LSPR for biosensing applications have been reported using different types and shapes of metal nano-structures [1–5]. Among the various nano-structures of noble metals, metal nano-islands (NIs) can be easily and reproducibly fabricated by conventional evaporation and heat treatment without any patterning processes. In addition, the relatively strong adhesion of NI films to a substrate gives the NI sensors mechanical robustness [2]. Previously, we have developed a novel approach for the detection of biomolecules, in which LSPR optical detection with gold NI was implemented to analyze binding of proteins to surfaces functionalized with the corresponding high affinity ligands. This method was used to rapidly detect recombinant GST-tagged hIL6 expressed in *Escherichia coli* by attenuated total reflection (ATR) image measurements [4]. In our previous study, the analyte molecules were directly captured by gold NI surfaces functionalized with small sized receptors such as biotin or glutathione molecules. In that case, we were able to observe a sufficient increase in the LSPR signal when the analyte molecules adsorbed to the gold NI, even at low analyte concentrations. However, when large molecules, such as proteins, are used as the receptors, the sensitivity in detecting binding events with LSPR is expected to be conspicuously lower. This is expected since the penetration depth of the LSP field in metal 3-D nanostructures is a few tens of nanometers at most [6–8]. In this study, we demonstrated a novel approach in which enzyme-catalyzed precipitation was induced on the gold nano-island (NI) surface after binding between interferon-γ (IFN-γ) and an IFN-γ antibody to enhance the sensitivity of detection based on LSPR analysis of gold NIs.

## 2. Results and Discussion

### 2.1. Annealing Effect on the Morphology and LSPR Spectrum of a Thin Gold Film

In general, comparatively thick gold films (t ≥ 15 nm) created with typical deposition rate (>1Å /sec) have a continuous morphology with slight roughness [9] and exhibit a minimum absorbance near 500 nm.

**Figure 1.**
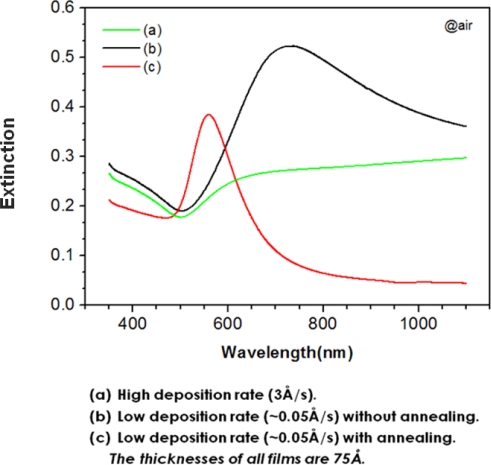
Extinction spectra of gold films obtained from different processes.

**Figure 2.**
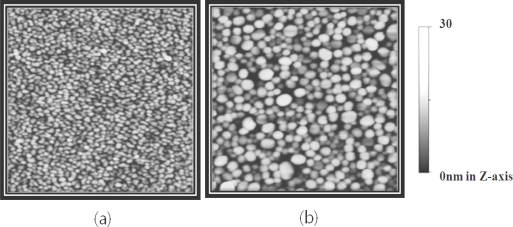
2-D AFM images of (a) non-annealed gold NI film and (b) annealed gold NI film. The scan ranges are 1 × 1 μm^2^.

Thin gold films (t < 10 nm) that are deposited slowly (<0.1 Å/sec) show an extinction maximum attributed to excitation of the localized surface plasmon (LSP) in the near IR range. After heat treatment of the intact gold NI film, the LSP band of the gold NIs shifts to the visible range and consequently, the extinction band appears near 560nm [10], similar to the extinction band of gold nanoparticles immobilized on transparent substrates [1,2] (Figure 1). The annealing effect on the extinction spectra of gold NI films originates from the changes in the morphology of the gold films. Figure 2 shows the AFM images of the gold films before and after heat treatment. The average height and diameter of the gold NI increased from 5.3 nm to 17.3 nm and from 29.5 nm to 67.2 nm, respectively, as a result of heat treatment. These results are in agreement with those of previous studies [4,10].

In the detection using LSPR, the peak shift or amplitude of the LSP band is generally measured as the local environment of metal nano structure is changing. In case of measuring the wavelength shift of the LSP band, the definition of the spectral centroid of the LSP band using a proper baseline is more effective than tracing its peak position [11]. The centroid (λ_cent._) of an extinction spectrum (ε(λ)) as a function of wavelength (λ) is given by

(1) $$\lambda_{\text{cent}.}=\frac{\int_{\lambda_{1}}^{\lambda_{2}}\lambda (\varepsilon (\lambda )-\varepsilon_{B})d\lambda }{\int_{\lambda_{1}}^{\lambda_{2}}(\varepsilon (\lambda )-\varepsilon_{B})d\lambda }$$

where ε_B_ is the baseline value, which is chosen as the half-maximum of the extinction spectrum, and λ_1_ and λ_2_ are the wavelengths at which the baseline intersects the extinction spectrum.

When the ambient medium for the bare NIs was changed from air to DI water, the change in the centroid position (Δλ_cent._) of the LSP band was 28 nm (the data not shown). The detection sensitivity of the refractive index with the gold NI array fabricated in this study was not largely different from the detection sensitivity reported in previous studies that have examined similar nano structures [1,2].

### 2.2. Signal Amplification using Enzymatic Precipitation in Immunoassay with Gold NI Chip

**Figure 3.**
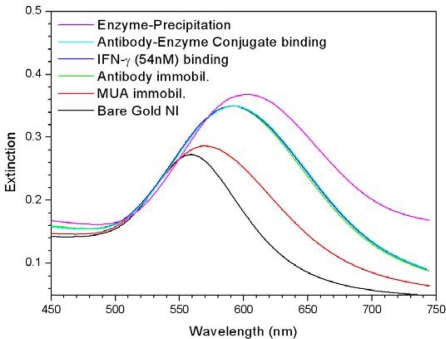
Changes in the extinction spectra of the gold NI film due to the modifications steps which proceed from bare film to immobilization of the antibody, to binding of IFN-γ antigen, to enzyme-catalyzed precipitation.

Figure 3 shows the LSPR spectra of gold NIs on a glass wafer measured after each step of the immobilization and binding process (described in the experimental section). As shown in this figure, the intensity of the maximum extinction increased and the λ_cent._ of LSPR band shifted to longer wavelengths after each subsequent modification step of the gold NI surface. This occurred because as the number of molecules adsorbed on gold NI surfaces increased, there was a corresponding increase in the dielectric constant of the local regions near the interface of the gold NIs. This was especially true when large antibody molecules (∼150 kDa, 0.1 mg/ml) were bound to the 11-mercaptoundecanoic acid (MUA) surfaces on the gold NI films, which also led to the change in the color of the gold NI films from purple to violet, as observed by the naked eyes. However, the bindings of *IFN-γ* (54 nM) as the analyte and of a large-sized antibody-enzyme conjugate induced only 0.25 nm and 0.33 nm of Δλ_cent._ in LSPR band, respectively. These changes are negligible responses, considering the resolution of the measuring instrument and that Δλ_cent._ values were 3.1 and 9.5 nm when MUA was immobilized on the bare NI surface and the when *IFN-γ* antibody was immobilized on MUA surface, respectively.

Consequently, it is impossible to detect 54 nM of *IFN-γ* in the sample solution using a label-free immunoassay with LSPR of the gold NI chip. This is inconsistent with the results of previous study [4], in which the binding of STA and recombinant GST-tagged protein molecules was able to be detected down to concentrations of a few nM with the gold NI surface. To explain the discrepancy between these results, the difference in the size of the receptor molecules bound to gold NI should be considered. In the previous study, the gold NI surface was functionalized with small receptors such as biotin and glutathione, which resulted in sufficient changes in the intensities of the LSPR bands of the gold NIs. In the present study, however, large antibodies (∼150 kDa) were used as receptors. Biosensors using LSPR are based on the fundamental principle that the changes in the amplitude and wavelength of the LSPR band originate from the increase in the dielectric constant near the metal NIs due to adsorption of analyte molecules to the NI surface and therefore, the exposure of the molecules to the plasmon field is a significant factor for sensitive detection of molecules.

In principle, the amplitude of the LSP field is attenuated at an extremely rapid rate in the direction normal to the surface of the metal; thus, the penetration depth of the LSP field into the ambient media in metal 3-D nanostructures is approximately 20 nm [8], which is about one tenth shorter than a propagating surface plasmon (PSP) field created in 2-D metal nano-films using a prism coupler [12,13]. As a result, the analyte molecules adsorbed on NI surfaces that have been functionalized with large antibodies (∼15 nm) and linkers (2∼3 nm) would not be extensively exposed to the LSP field. Therefore, we can expect that the sensitivity in detecting intermolecular binding events when using metal NIs with large antibodies would be conspicuously lower than when using small receptors. In fact, this result is consistent with a previous investigation [2], in which the absorbance increased by only a slight degree even though a great deal of HSA was bound to gold NPs modified with the anti-HSA (∼150 kDa) on quartz substrates.

To enhance the sensitivity of biomolecular sensing using gold NI films, we tried to amplify the signal change of LSPR in combination with a precipitation scheme induced by an enzymatic reaction on the gold NI surface. As a result, the centroid of the LSPR band shifted from 592.2 nm to 603.7 nm after the precipitation of 4-CN induced by HRP conjugated with the antibody. This resulted in a Δλ_cent._ of 11.5 nm, which led to an amplification of the signal change after the specific binding of *IFN-γ* to the gold NI surface (Figure 3).

**Figure 4.**
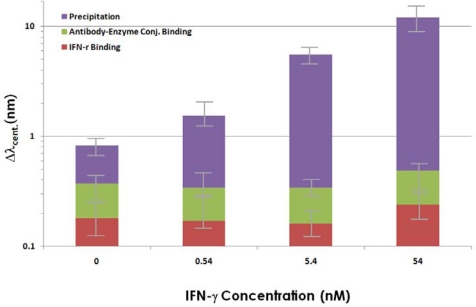
Centroid shifts in the LSPR band after various concentrations of *IFN-γ* were bound to anti-*IFN-γ* 5 and after the enzyme-catalyzed precipitation reactions, which significantly amplified the signal change. Each plotted value was averaged from the five identical experiments using different NI chips for each concentration and the error bars represent the standard deviations.

Different concentrations of *IFN-γ* were specifically adsorbed to the antibody immobilized on the Nis, and subsequently, the enzyme-catalyzed precipitations were induced, the results of analyzing the Δλ_cent._ for the LSPR spectra of gold NIs are summarized in Figure 4. As shown in the plot, no significant Δλ_cent._ of the LSPR band was observed over the range of *IFN-γ* concentrations used in this study, compared to the signal of the negative control (0nM of *IFN-γ*), when the precipitation reaction was not used. On the other hand, the precipitation reaction was found to dramatically increase the Δλ_cent._ for the whole range of *IFN-γ* concentrations. This effect was so profound that even the binding of 0.54 nM (total 3.25 × 10^10^ molecules) *IFN-γ* was detected. This was also more sensitive than when the amount of precipitation on glass slides that did not contain gold NIs was measured by absorption spectroscopy, which had an LOD (the limit of detection) of about 2 nM under identical conditions (the data not shown). It can be predicted that the increase in the Δλ_cent._ after precipitation resulted from the large increase in the local dielectric constant near the gold NIs, which is due to the adsorption of the dense precipitates composed of complexes containing a benzene ring and chlorine that have a high polarizability on the NIs. Even though the sensitivity of LSPR detection was enhanced using the precipitation, the sensitivity of the detection of *IFN-γ* was still lower than that found in the previous study [14], where the same protein was detected using SPR and SPR imaging. This discrepancy can be attributed the inherent low sensitivity of the LSPR detection method originating from the shallower penetration depth of the plasmon field in LSPR than in SPR. Moreover, the metal NIs fabricated using evaporation and heat treatment exhibit random distributions in their sizes and locations unlike the ordered arrays of metal nanodots obtained by the nanolithography [6]. Therefore, the LSPR spectrum of the random metal NIs is the ensemble average of a number of NIs observed in the experiment, which seems to result in the deterioration of the detection sensitivity due to spectral broadening. We expect that the detection sensitivity would be enhanced by about ten-fold if ordered nanodot arrays were used instead of random NIs.

## 3. Experimental Section

### 3.1. Formation of Fold Nano-Island(NI) Films on Glass Substrates

The formation of gold NIs on glass substrates was previously described [4]. First, a glass wafer was cut into several square chips (5 × 5 mm^2^), cleaned in Piranha solution (concentrated H_2_SO_4_/ 30% H_2_O_2_, 4:1 v/v) and then rinsed with deionized (DI) water. *CAUTION: Piranha solution reacts violently with most organic materials and must be handled with extreme care.* Clean glass chips were treated with 3-mercaptopropyl trimethoxysilane instead of transition metals to increase the adhesive strength between the gold film and the glass surfaces without damping the LSPR in the gold NIs. The modified glass chips were coated with gold films using an electron beam evaporator. The gold films were deposited on the glasses at an average deposition rate of ∼0.1 Å/s under ∼1 × 10^−6^ torr.. The final thickness of gold film was 60 Å. The glass chips coated with gold films were heat-treated at 210 °C for 60 h in an N_2_-atmospheric furnace. The gold NI chips were then treated with a solution containing a 1:1 volume ratio of CH_2_Cl_2_ and ethanol to stabilize the chips against the blue shift of the plasmon band, which typically appears in metal nano-islands immersed in solvents [10].

### 3.2. Modifications of Gold NI Surfaces and Immobilization of IFN-γ antibody on Gold NI Surfaces

**Figure 5.**
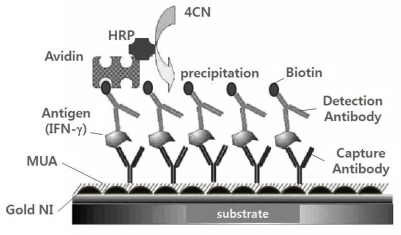
Schematic illustration of the sandwich-type immunoassay employed in this study. For simplicity, the components are not drawn to scale.

The gold NI surfaces were immersed in 10 mM 11-mercaptoundecanoic acid (MUA: Sigma-Aldrich, USA) solution in ethanol for 12 h at 25 °C. The schematic illustration of the immunoassay method employed in the present study is shown in Figure 5. The self assembled MUA monolayer on the gold surface was activated by transformation into a hydroxysuccinimidyl ester by incubating the chip with a mixture of *N*-hydroxysuccinimide (NHS, 0.05 M) and 1-ethyl-3-(3-dimethylaminopropyl) carbodiimide hydrochloride (EDAC, 0.2 M) in DI water. The activated chip was then washed with DI water and dried under a mild stream of N_2_. To immobilize the antibodies on the activated MUA surface, the chips were incubated in phosphate-buffered saline (PBS: pH 7.4) containing 0.1 mg/mL of anti-IFN-γ for 1 h. Non-specific binding was prevented by using ethanolamine and bovine serum albumin (BSA; 1 mg/mL). The chips were reacted in a PBS solution that contained different concentrations of recombinant IFN-γ and 1 mg/ml BSA for 1 h. Each immobilization and binding reaction was performed in a petri-dish that was hermetically sealed to prevent the solution from evaporating.

### 3.3. Signal Amplification Using an Enzymatic Precipitating Reaction on the Gold NI Surface

After thorough washing with PBS to remove nonspecifically bound IFN-γ, the chip was incubated with a mixture of biotinylated IFN-γ detection antibody of and avidin-HRP (20 μg/mL each). Finally, to amplify the signal with the immunoprecipitation reaction, a reaction solution containing H_2_O_2_ (1 mM) and 4-CN (1mM) was added to the chip’s surface. Consequently, an HRP mediated conversion (10 min) of 4-CN to benzo-4-chlorocyclohexadienone with H_2_O_2_ yielded an insoluble precipitate on the sensor surface where the biocatalyzed reaction took place [15].

### 3.4. Measurements of LSP Bands and Surface Morphologies

The changes in LSPR spectra of gold NIs resulting from the modifications of the gold NI surface, the binding of IFN-γ and the enzyme-catalyzed precipitation were observed under atmospheric conditions using a DU 800 spectrophotometer (Beckman-Coulter, USA) with an optical resolution of 0.25 nm. The surface morphologies of the gold NI films before and after heat treatment were imaged with an atomic force microscope (AFM: Digital Instruments, USA) in non-contact mode.

## 4. Conclusions

We demonstrated the feasibility of using gold NI fabricated via vacuum evaporation as a sensing surface for LSPR-based detection applications, such as the development of biosensors and biochips. In the present experiment on biomolecular detections based on the LSPR of the gold NI surface, the precipitation of 4-CN on the gold NI surface, which was catalyzed by HRP, was found to greatly enhance the sensitivity of detecting binding events between large antibody receptors and biomolecules. Consequently, we were able to successfully detect IFN-γ binding at a concentration of 0.54 nM in PBS using the precipitation reaction; a limit of detection was 100 times lower than the LOD of the detection without the enzymatic reaction.
